# Potential of *Medicago sativa* and *Perilla frutescens* for overcoming the soil sickness caused by ginseng cultivation

**DOI:** 10.3389/fmicb.2023.1134331

**Published:** 2023-04-05

**Authors:** Xingbo Bian, Xiaohang Yang, Kexin Zhang, Yiru Zhai, Qiong Li, Lianxue Zhang, Xin Sun

**Affiliations:** ^1^College of Pharmacy, Jilin Medical University, Jilin, China; ^2^Jilin Ginseng Academy, Changchun University of Chinese Medicine, Changchun, China; ^3^College of Chinese Medicinal Materials, Jilin Agriculture University, Changchun, China

**Keywords:** ginseng cultivation soil, soil sickness, soil microbial community, alfalfa, perilla

## Abstract

There are serious soil sickness in ginseng cultivation. Crop rotation is an effective agricultural management to improve soil sustainability and reduce soil sickness. To explore an appropriate ginseng rotation system, *Medicago sativa* (alfalfa) and *Perilla frutescens* (perilla) were planted on ginseng cultivation soil for 1 year to evaluate the improvement effect of both. Through chemical analysis and high-throughput sequencing technology, we found that after alfalfa and perilla cultivation for one-year, various nutrients and enzyme activities in ginseng cultivation soil were significantly improved. In addition, perilla significantly increased the diversity and richness of soil fungal communities. Cultivation of alfalfa and perilla significantly changed the composition of soil bacterial and fungal communities and significantly reduced the abundance of the potentially pathogenic fungi *Ilyonectria*. Further pot experiments also showed that the improved soil could significantly increase root activity of ginseng plant after two plants were planted. It should be noted that, unlike alfalfa, perilla decreased soil electrical conductivity, increased soil organic matter, soil urease, and may significantly improve the diversity and richness of soil fungal community. Moreover, in the pot experiment, the root fresh weight of ginseng cultured in perilla treated soil increased significantly. This study highlights that perilla may have better soil improvement effect than alfalfa and it has the potential to be used in the soil improvement of ginseng cultivation.

## Introduction

Ginseng (*Panax ginseng* Meyer) is one of the most famous Chinese herbal medicine in China with a history of thousands of years (Xiang et al., 2008; Yun, 2009; Yang et al., 2017). Ginseng is now widely cultivated in northeast China, Korea, and Japan (Baeg and So, 2013; Ru et al., 2015). Artificial cultivation of ginseng usually takes 4 to 6 years and requires extremely high soil quality (Proctor and Bailey, 1987). More importantly, due to allelopathy and soil fertility changes, ginseng is subject to substantial soil sickness, which seriously affects the yield of ginseng (Xiao et al., 2016). After ginseng has been planted, the soil is often called “old ginseng soil.” In this soil, it is difficult for ginseng to grow again and difficult for many other crops to grow normally, which affects the utilization rate of land. So far, the mechanism underlying soil sickness of ginseng is not completely clear. Scholars believe that the occurrence of soil sickness may be related to the effect of ginseng exudates on soil’s physical and chemical properties and microbial community (Zhang and Xue, 2010; Li Q. et al., 2021).

In fact, research has proven that farming systems relying on crop rotation to improve plant nutrition and control weeds, pests and plant pathogens are an ancient but attractive and environmentally friendly approach (Altieri, 2018). Implementing scientific crop rotation or effective soil improvement methods is the most effective measure to reduce or eliminate soil sickness (Li C. et al., 2021). The underlying mechanisms include improving soil physicochemical properties, increasing the diversity of soil microbiota, and associated beneficial microorganisms, and breaking the natural life cycle of soil-borne and airborne pathogens, as well as reducing the harmful effects of autotoxic compounds released by crops (Mäder et al., 2002; Peters et al., 2003; Gong et al., 2021; Woo et al., 2022). Therefore, it has become vital to find one or several crops that can generally grow after ginseng cultivation and positively impact the soil’s physicochemical properties and microbial community. Furthermore, the relevant research is of great significance to the development of the ginseng crop rotation system and the improvement of ginseng cultivation soil. Perilla (*Perilla frutescens* L.) is widely used as medicine and food in various Asian countries (Ahmed, 2019; Wei et al., 2022). It is also an adaptive and large biomass plant (Xiao et al., 2020). In previous studies, American ginseng (*Panax quinquefolium* L.)-perilla rotation could effectively resolve the continuous cropping problem of American ginseng (Zhao et al., 2005). Recent studies have found that perilla has a good potential in dealing with environmental pollution (Shu et al., 2021). The legume alfalfa (*Medicago sativa* L.) is a “microbial hotspot crop” (Baslam et al., 2012). Cultivation of alfalfa produces a large amount of root biomass, generating root exudates that recruit soil bacterial populations to the rhizosphere, so it is often used in crop rotations (Tesfaye et al., 2003; Samaddar et al., 2021). Previous studies have shown that alfalfa fixes atmosphere N_2_ to reduce or eliminate the need for synthetic N fertilizer and effectively increase crop yields in crop rotations (Fernandez et al., 2019). More importantly, perilla and alfalfa often grow in wild or semi-wild forms in ginseng fields. Therefore, these two plants may not be sensitive to the allelopathic effect produced by ginseng. At present, the effects of planting other crops on soil fertility, physicochemical properties, and microbial communities of soils after ginseng cultivation are not clear. In addition, there are no reports on the application of perilla or alfalfa to improve the soil after ginseng cultivation.

In this context, it is reasonable to speculate that the cultivation of perilla or alfalfa may improve the soil quality of ginseng cultivation. Therefore, in this study, perilla and alfalfa were used, respectively, to conduct one-year planting experiments on ginseng cultivation soil. Moreover, physical and chemical properties and microbial communities in perilla and alfalfa cultivated soils were analyzed, and compared with that in the untreated ginseng cultivation soil. Through this work, we will achieve the following objectives: (a) to compare the soil fertility and bacterial and fungal community composition among ginseng cultivation soil planted with perilla for 1 year, ginseng cultivation soil planted with alfalfa for 1 year, and untreated ginseng cultivation soil; (b) to analyze the improvement effect of two crops, perilla and alfalfa, on ginseng cultivation soil, and to provide data and theoretical basis for ginseng cultivation soil improvement and ginseng crop rotation strategy.

## Materials and methods

### Study site and experimental design

The field experiment was carried out on an experimental farm in Choushui Village, Fusong County, Jilin Province, China (42.40′ N and 127.09′ E). Fusong region is the production area of genuine ginseng medicinal materials and is known as the hometown of ginseng in China (Liu et al., 2011). In detail, the experimental site of this study has a temperate monsoon climate at an altitude of 410 m. The average annual temperature is 4°C, with an average temperature of −33°C in January and 30°C in July. The average annual frost-free period is 120 days, and the average annual precipitation is 800 mm.

The experiment field was cultivated for 4 years from 2017 to 2020, and the ginseng was taken out in 2020. The whole process of ginseng cultivation strictly follows the local standards of “ginseng safe production technical specification of pesticide application (DB22/T 1233–2019).”

The field where ginseng had been cultivated was then divided into 3 groups with 4 plots in each group: (1) no treatment (i.e., control check; *n* = 4); (2) perilla cultivation (*n* = 4); (3) alfalfa cultivation (*n* = 4). There are 12 plots in total, each with an area of 10 m^2^ and with distance of 0.5 m between adjacent plots. In addition, the same group of plots are not directly adjacent (detailed plots distribution is shown in Supplementary Figure S1). Perilla and alfalfa were cultivated for 1 year (from August 2020 to August 2021). The seeds of *Perilla frutescens* Britt. var. *frutescens* and *Medicago sativa* subsp. *falcata* were purchased from Changjingyuanlin Co. LTD (Jiangsu, China). Simply put, after the field was prepared, the seeds were sown. Considering production requirements, the growth density of perilla is about 5 plants per square meter, and that of alfalfa is about 150 plants per square meter. All crops were planted simultaneously and irrigated (it is usually watered every 5 days to keep the soil moist and drained when it rains more) to ensure their survival during the beginning of the growing period. Moreover, all experiment plots were not treated with fertilizer or other manual interventions except for regular weed cleaning.

### Samples selection and collection

Samples of soil were collected from each plot 1 year after planting on the same day, 5 August 2021. Sampling was carried out based on the above experimental design. In each plot, five subsamples were randomly collected at a depth of 0–20 cm, which were mixed into one soil sample. Therefore, we finally obtained four soil samples from each treatment. After all soil samples were passed through a 2 mm sieve, part of each sample was frozen at −80°C for DNA extraction. The other part was air-dried at room temperature to determine chemical properties and enzyme activities.

### Soil sample analyses

#### Characterization of soil chemical properties

The soil pH of all samples was determined (soil-water ratio of 1:2.5) by using a pH meter. Moreover, soil electrical conductivity (EC) was determined by a conductivity meter (soil-water ratio of 1:5). The potassium dichromate oxidation-external heating method was used to measure soil organic matter (Fan et al., 2017). Soil available nitrogen was analyzed using the alkaline hydrolysis method (Gianello and Bremner, 1986). Soil available phosphorus was determined by the NaHCO_3_ extraction molybdenum antimony colorimetry method (Olsen, 1954). Soil available potassium was determined by the NH_4_OAc extraction fame photometric method (Yan et al., 2021b).

#### Analysis of soil enzyme activities

The soil urease, acid phosphatase, sucrase, catalase, and laccase activities were determined using the soil enzyme kit from Solarbio Science and Technology Co. (Beijing, China). Briefly, the soil urease assay was performed with method of indophenol blue colorimetry; the determination of acid phosphatase was based on the disodium phenyl phosphate colorimetry; sucrase activity was estimated by the 3,5-dinitrosalicylic acid colorimetry; the activity of catalase was determined by KMnO_4_ titration; the laccase activity was determined by ABTS colorimetry.

#### Microbial community analysis

##### DNA extraction and PCR amplification

The bacterial and fungal DNA from 0.5 g of soil was extracted in triplicate using A.E.Z.N.A. Soil DNA Kit (Omega Bio-tek Inc., United States), followed by agarose gel electrophoresis to detect the quality of DNA (Pan et al., 2020). Then, the DNA concentration was determined by NanoDrop (2000) UV–vis spectrophotometer (Thermo Scientific, United States). To identify bacteria, the V3–V4 regions primers 338F (50-ACTCCTACGGGAGGCAGCAG-30) and 806R (50-GGACTACHVGGGTWTCTAAT-30) were selected to amplify the soil bacterial 16S rRNA gene (Zhang et al., 2020). For fungi, the Internal Transcribed Spacer (ITS) regions of the fungal gene were amplified with the primers ITS1F (5′-CTTGGTCATTTAGAGG AAGTAA-3′)—ITS2R (5′-GCTGCGTTCTTCATCGATGC-3′; Liang et al., 2021). The PCR amplification was performed as follows: 95°C for 3 min, 95°C for 30 s, 55°C for 30 s, and 72°C for 45 s (27 cycles for bacteria and 35 cycles for fungi), followed by 10 min at 72°C.

##### Illumina MiSeq sequencing analyses

The PCR products were purified by an AxyPrep DNA Gel Extraction Kit (Axygen Biosciences, Union City, CA, United States) and quantified by the quantitative analyzer™ Fluorometer (Promega, United States). All purified PCR products were used for Illumina MiSeq high-throughput sequencing with Illumina’s MiSeq PE300 platform at the Majorbio Bio-pharm Technology Co., Ltd. (Shanghai, China). The raw sequencing data have been uploaded to the NCBI Sequence Read Archive database (Bacterial Accession Number: PRJNA820351; Fungal Accession Number: PRJNA820493).

##### Bioinformatic analysis for sequencing data

Paired-end reads were assigned to samples based on their unique barcode and truncated by cutting off the barcode and primer sequence. Raw reads were filtered to obtain high-quality reads with the QIIME1 software (version 1.9.1, http://qiime.org/install/index; Caporaso et al., 2010). The high-quality reads with >97% sequence similarity were classified into different operational taxonomic units (OTUs) using UPARSE (version 7.1, http://www.drive5.com/uparse/; Edgar, 2013). The taxonomy of each bacterial and fungal gene sequence was analyzed using the Ribosomal Database Project (RDP) Classifier (version 2.11, http://rdp.cme.msu.edu/) against the Silva128 16S rRNA database^1^ and Unite 7.0 ITS fungi database,^2^ respectively, with a 70% confidence threshold (Cole et al., 2009; Pan et al., 2020).

Soil bacterial functions were predicted using the PICRUSt tool based on 16S rRNA sequencing data, and biological functions were annotated in the KEGG database (Kanehisa and Goto, 2000; Langille et al., 2013; Gong et al., 2021). In addition, the FUNGuild tool was used for the functional prediction and classification of soil fungi (Nguyen et al., 2016; Schmidt et al., 2019).

### Pot experiment design

To further verify the improvement effect of alfalfa and perilla cultivation on ginseng cultivation soil, a pot experiment was conducted. After the one-year field experiment was finished, 20 kg of soil were collected treatment (no treatment, alfalfa, and perilla cultured treatments) for the pot experiment. To put it simply, a total of 18 polypropylene pots (diameter 24 cm, height 15 cm) were divided into 3 groups, with 6 pots in each group. Each group was loaded with corresponding soil collected in the field, 2,500 g of soil in each pot. Then, 2-year-old ginseng seedlings with similar root development and growth were selected for cleaning, and their roots were disinfected with sodium hypochlorite (Sun et al., 2018). Three ginseng seedlings were planted in each pot for 6 weeks. Watering and weeding were applied regularly.

After 6 weeks of growth, ginseng plant was collected and the aboveground part was removed from the stem base to obtain ginseng roots. The biomass (fresh weight and dry weight) of each group was measured. In addition, the root activity was determined by the 2,3,5-triphenyl tetrazolium chloride (TTC) method (Zhan et al., 2021). Root activity is expressed as the mass of triphenyl formazan produced per gram of fresh root per hour.

### Statistical analysis

Based on OTUs, the alpha diversity of microbial communities was quantified, including richness (ACE and Chao1) and diversity (Shannon and Simpson) indexes. Beta-diversity was used for comparative analysis of microbial community composition in different groups. Principal co-ordinates analysis (PCoA) and redundancy analysis (RDA) was conducted using the R software (version 4.1.0, https://cran.r-project.org/; Dixon, 2003). LEfSe analysis (Linear Discriminant Analysis Effect Size) was used to elucidate the biomarkers in each group.

Data on soil physical and chemical properties, enzyme activities, and soil microbial alpha diversity were analyzed using the SPSS software (IBM Corporation, Armonk, NY, United States). The results were expressed as the arithmetic mean value ± standard deviation. The differences in the means were compared by the One-way ANOVA at *p* < 0.05.

## Results

### Soil properties

**Figure 1:**
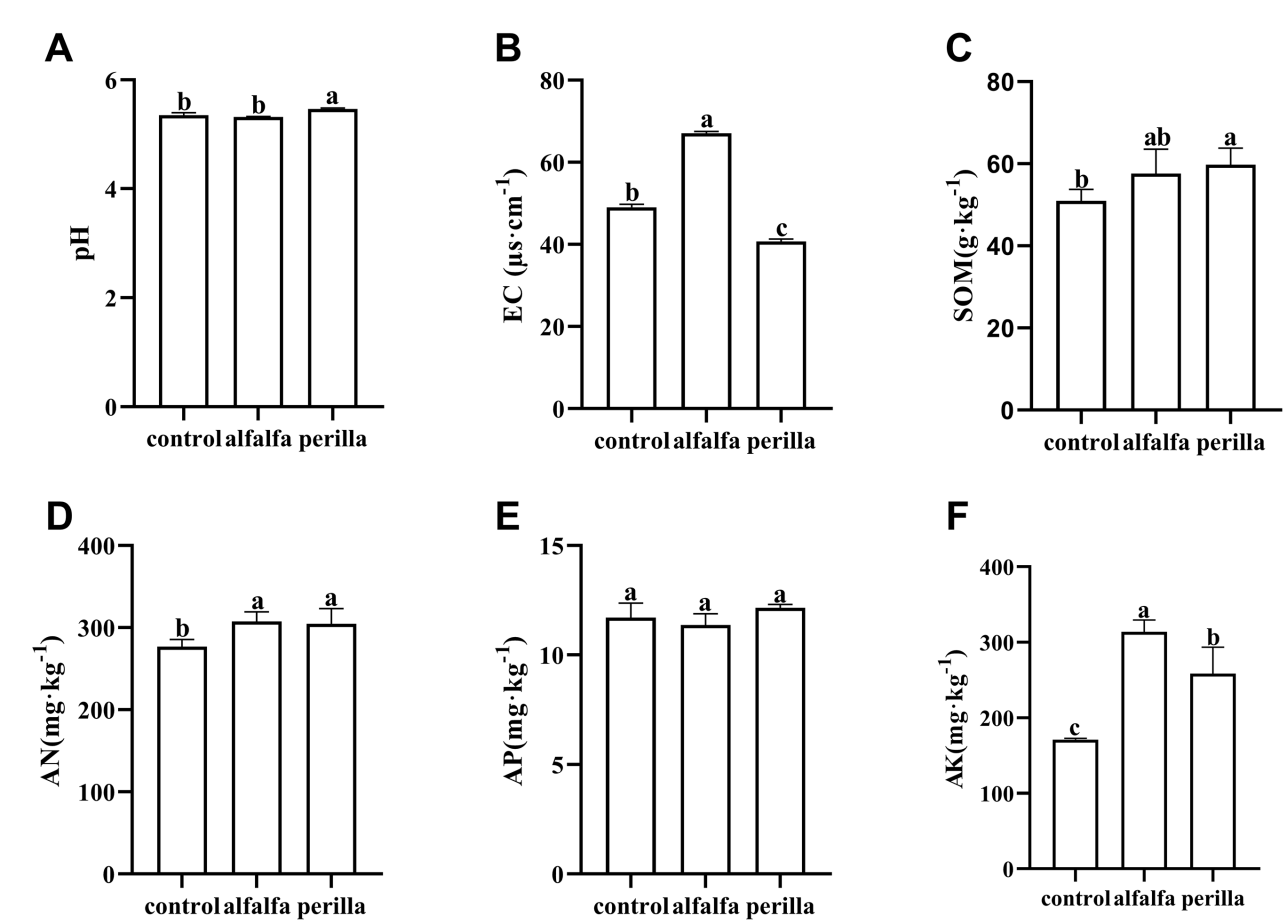
Physicochemical properties and nutrient content of the soil in different treatments. Control, no treatment; Alfalfa, alfalfa cultivation; Perilla, perilla cultivation. **(A)** pH; **(B)** EC, electric conductivity; **(C)** SOM, soil organic matter; **(D)** AN, available nitrogen; **(E)** AP, available phosphorus; **(F)** AK, available potassium. Error represents the standard deviation; different letters indicate a significant difference at the *p* < 0.05 level.

Analysis of soil properties showed that pH of the perilla group was significantly higher than that of the control group, while the alfalfa group showed no significant change (Figure 1A). The EC values in the perilla group were significantly lower than those in the control group; in contrast, the EC values in the alfalfa group were significantly higher than those in the control group (Figure 1B). Soil organic matter was significantly higher in the perilla group than that in the control group (Figure 1C). Soil available nitrogen and available potassium were significantly higher in both the perilla and alfalfa groups than that in the control group (Figures 1D,F). And, available potassium was significantly higher in the alfalfa group than that in the perilla group (Figure 1F). Furthermore, there was no significant difference in available phosphorus among all the groups (Figure 1E).

### Soil enzyme activities

**Figure 2:**
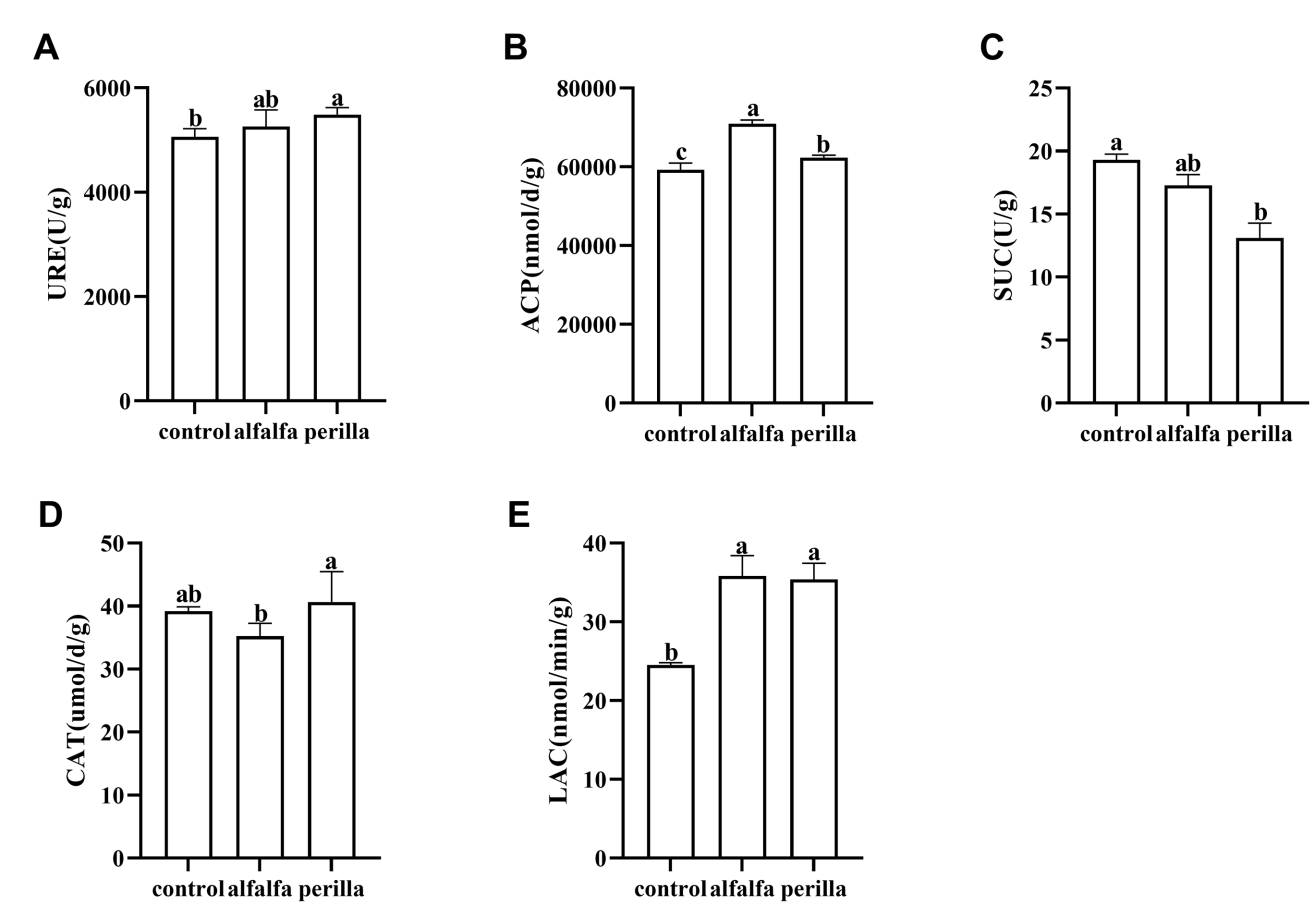
Enzyme activities of the soil in different treatments. Control, no treatment; Alfalfa, alfalfa cultivation; perilla, perilla cultivation. **(A)** URE, urease; **(B)** ACP, acid phosphatase; **(C)** SUC, sucrase; **(D)** CAT, catalase; **(E)** LAC, laccase. Error represents the standard deviation; different letters indicate a significant difference at the *p* < 0.05 level.

The soil urease activity in the perilla group was significantly higher than that in the control group (Figure 2A). In addition, the acid phosphatase and laccase activities in both the perilla and alfalfa groups were significantly higher than those in the control group, and the acid phosphatase activity in alfalfa was also significantly higher than that in the perilla group (Figures 2B,E). In contrast, the sucrase activity in the perilla group was significantly lower than that in the control group (Figure 2C). The catalase activity of the perilla group was significantly higher than that of the alfalfa group. In contrast, the catalase activity of these two groups was not significantly different from that of the control group (Figure 2D).

### Microbial diversity and richness

**Figure 3:**
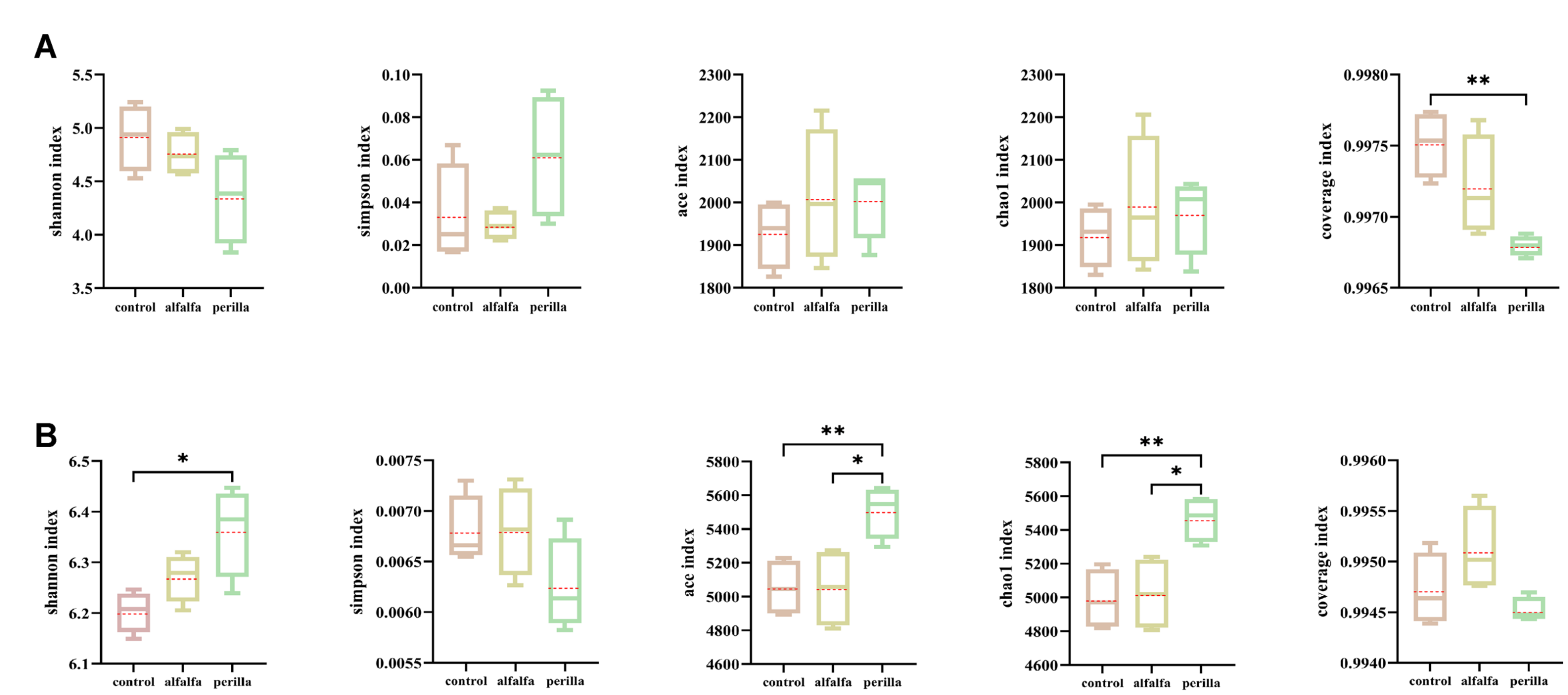
Soil **(A)** bacterial and **(B)** fungal alpha diversity in different treatments. Control, no treatment; Alfalfa, alfalfa cultivation; Perilla, perilla cultivation. “*“and “**” indicate a significant difference at the *p* < 0.05 and *p* < 0.01 level, respectively.

There were no significant differences in Shannon, Simpson, ACE, and Chao1 indexes of bacteria among different treatments. Furthermore, the coverage index of each group was more than 0.996 (Figure 3A). In terms of fungi, the Shannon index was significantly higher in the perilla group than that in the control group, while the Simpson index was not significantly different among the groups. Both ace and chao1 indexes were significantly higher in the perilla group than that in the control and alfalfa groups. In addition, the coverage indexes of the three groups were all greater than 0.994 (Figure 3B). Overall, alfalfa cultivation had no significant effect on the diversity and richness of microbial communities in ginseng cultivation soils. In contrast, the cultivation of perilla increased the diversity and richness of fungal communities in ginseng cultivation soils.

### Microbial community composition and structure

**Figure 4:**
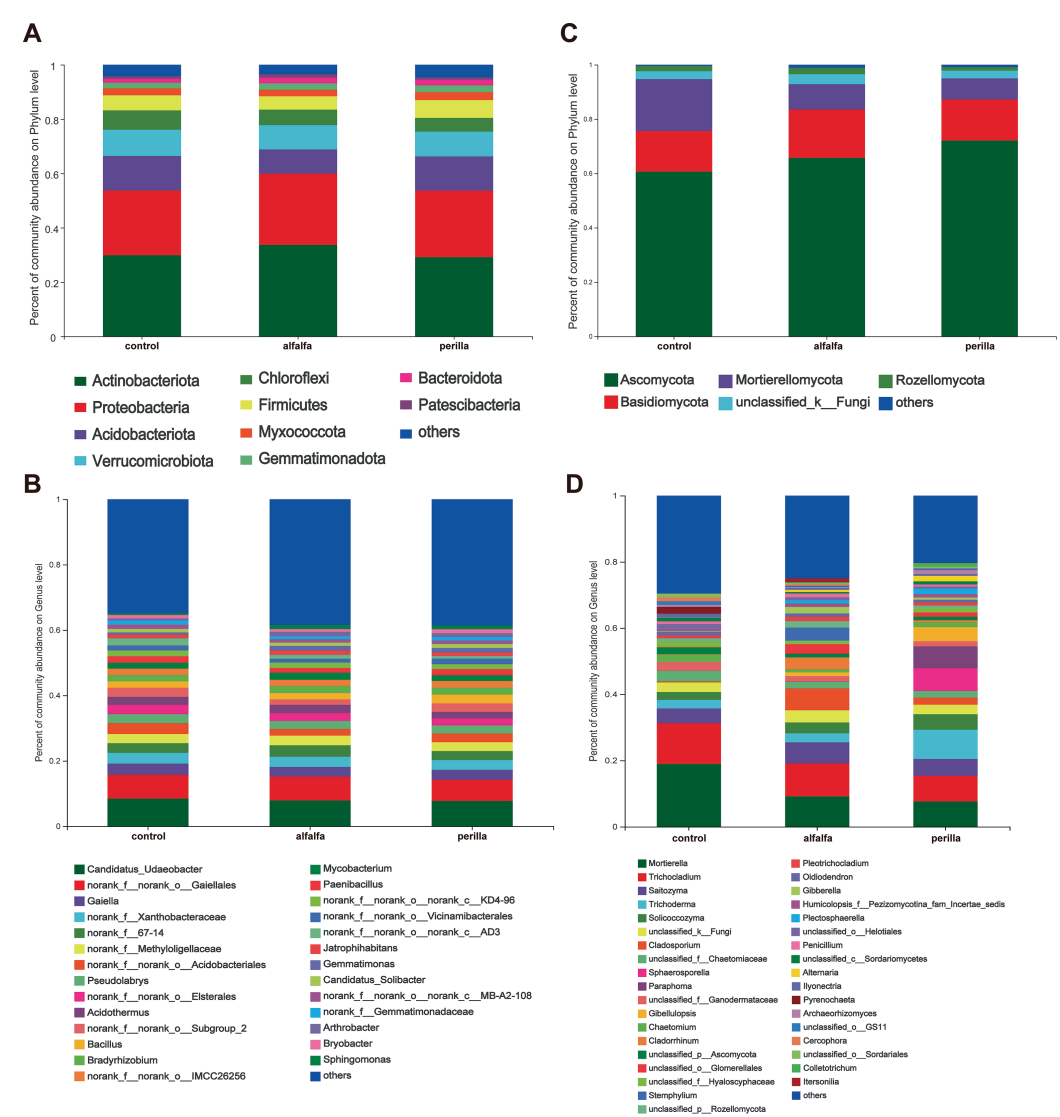
Relative abundances of **(A)** bacterial and **(B)** fungal phylum level and **(C)** bacterial and **(D)** fungal genus level in different treatments. Control, no treatment; Alfalfa, alfalfa cultivation; Perilla, perilla cultivation.

The relative abundance of bacterial and fungal communities at the phylum level and genus level were counted for each group. The relative abundance at the bacterial phylum level showed that *Actinobacteria* were the dominant phylum, followed by *Proteobacteria* and *Acidobacteriota* (Figure 4A). In addition, we found that *Actinobacteria* and *Proteobacteria* were significantly higher in the alfalfa group than that in the other two groups. On the contrary, the abundance of *Acidobacteriota* in the alfalfa group was significantly lower than that in the other two groups (Supplementary Figure S2). The most abundant bacterial at the genus level were *Candidatus Udaeobacter*, followed by norank-o-*Gaiellales* and *Gaiella* (Figure 4C). For fungi, the highest relative abundance at the phylum level was found in *Ascomycota*, followed by *Basidiomycota* and *Mortierellomycota* (Figure 4B). The *Mortierellomycota* abundance in the alfalfa and perilla groups was significantly lower than that in the control group (Supplementary Figure S3). The fungal genus with the highest relative abundance was *Mortierella*, followed by *Trichocladium* and *Saitozyma* (Figure 4D). We also found that the abundance of a common ginseng pathogen genus, *Ilyonectria*, was significantly lower in the alfalfa and perilla groups than that in the control group (Supplementary Figure S4).

**Figure 5:**
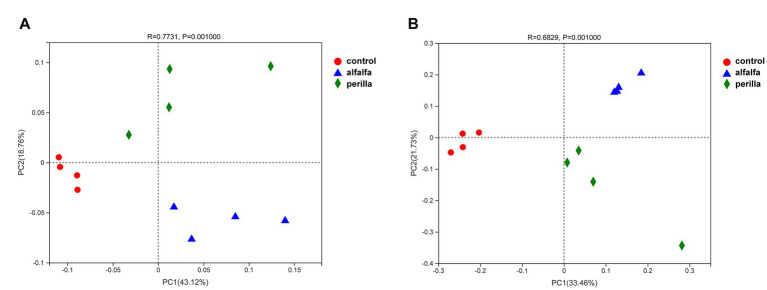
PCoA of **(A)** bacterial and **(B)** fungal communities in different treatments. Control, no treatment; Alfalfa, alfalfa cultivation; Perilla, perilla cultivation.

PCoA and ANOSIM at the OTU level clearly distinguished between soil bacterial (R = 0.773, *p* = 0.001) and fungal (R = 0.683, *p* = 0.001) communities in different groups (Figure 5). The first two axes (PC1 and PC2) explained 43.12% and 18.76% in the bacterial community, respectively (Figure 5A). The PCoA of the fungal community was variance explained by PC1 and PC2 for 33.46% and 21.73%, respectively (Figure 5B).

### Indicator microbes for each group

**Figure 6:**
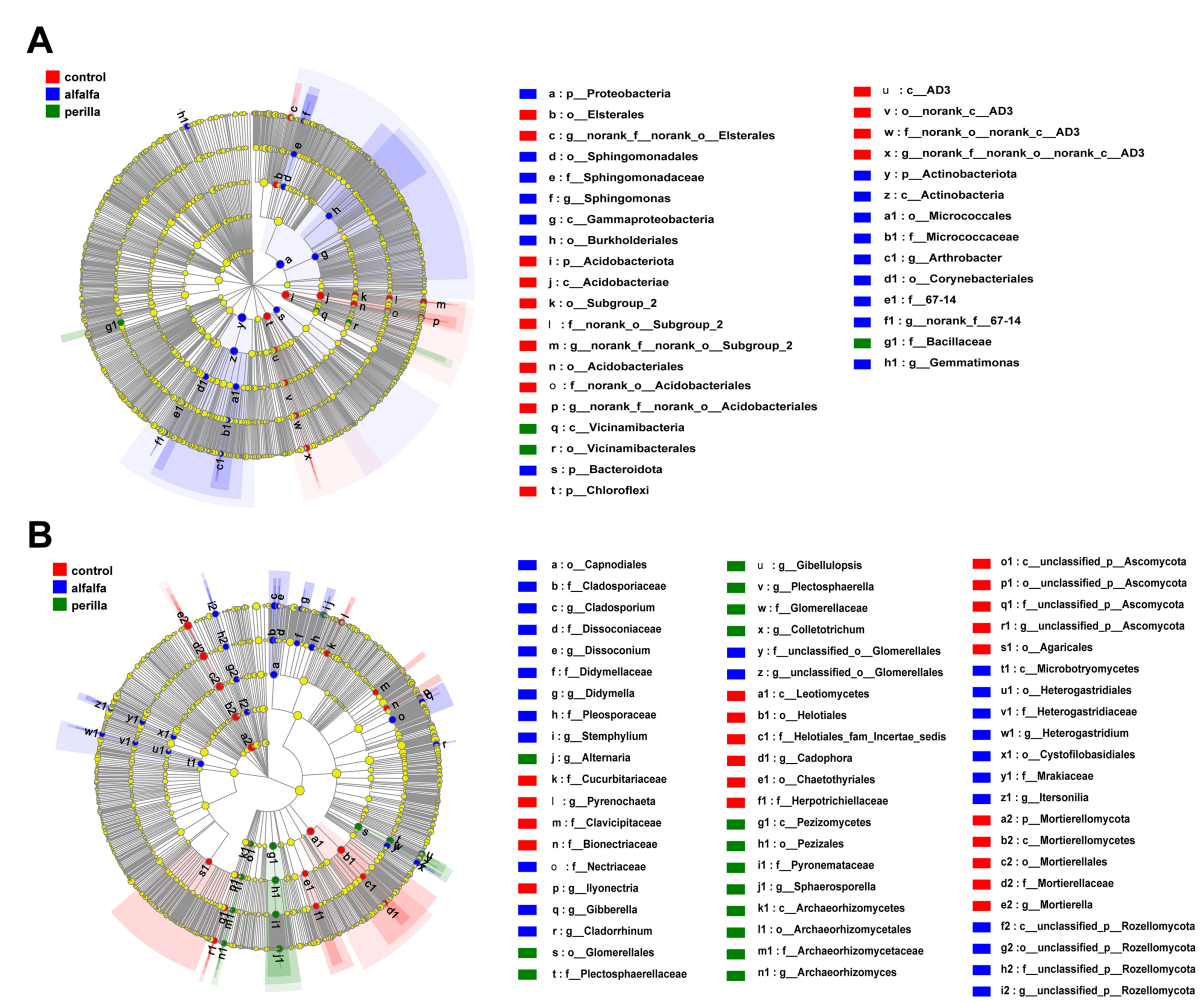
LEfSe analysis of **(A)** bacterial and **(B)** fungal communities in different treatments. Control, no treatment; Alfalfa, alfalfa cultivation; Perilla, perilla cultivation.

The Lefse tool analyzed indicator microbes in each microbial community group. As shown in Figure 6A, the indicator bacteria of the control group are the order *Elsterales*, the phylum *Acidobacteriota* (including the orders *Subgroup 2* and *Acidobacteriales*), and the phylum *Chloroflexi* (including the class *AD3*). The indicator bacteria of the alfalfa group are the phylum *Proteobacteria* (including the orders *Sphingomonadales* and *Burkholderiales*), the phylum *Bacteroidota* and the phylum *Actinobacteriota* (including the orders *Micrococcales* and *Corynebacteriales*). Furthermore, the indicator bacterial taxa of the perilla group are the family *Bacillaceae* and the order *Vicinamibacteria* (including the order *Vicinamibacterales*; Figure 6A).

In the Lefse analysis of fungal, the indicator taxa of the control group are the phylum *Mortierellomycota*, the phylum *Leotiomycetes* (including the orders *Helotiales* and *Chaetothyriales*), the order *Agaricales*, and the families *Cucurbitariaceae*, *Clavicipitaceae*, and *Bionectriaceae*. The indicator fungi of the alfalfa group are the phylum *Microbotryomycetes* (including the orders *Heterogastridiales* and *Cystofilobasidiales*), the order *Capnodiales* (including the families *Cladosporiaceae* and *Dissoconiaceae*), and the families *Didymellaceae*, *Pleosporaceae*, and *Nectriaceae*. The indicator fungi of the perilla group are the classes *Pezizomycetes*, *Archaeorhizomycetes*, and the order *Glomerellales* (including the genera *Gibellulopsis* and *Plectosphaerella*; Figure 6B). The LDA results for each group of bacterial and fungal communities are shown in Supplementary Figures S5A,B, respectively.

### Relationship between microbial community structure and soil properties

**Figure 7:**
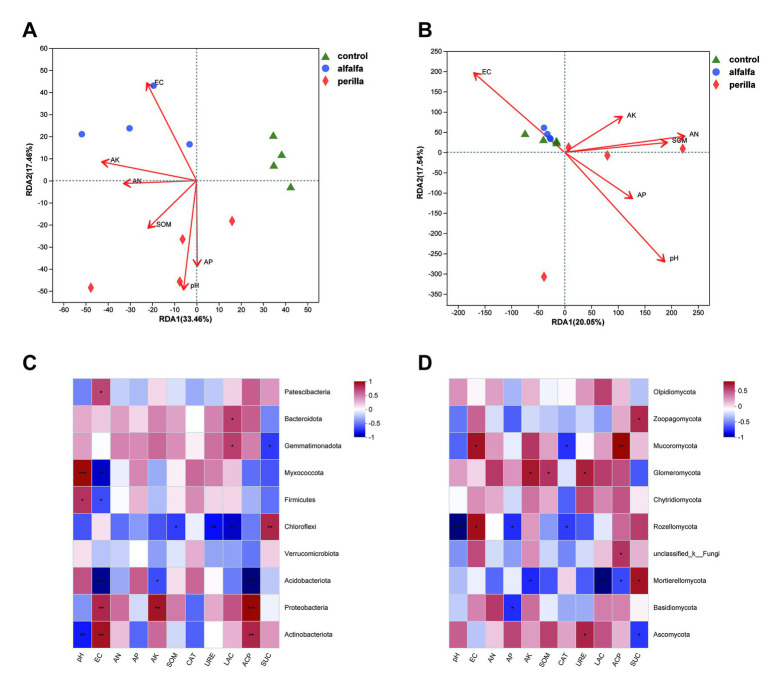
RDA analysis of **(A)** bacterial and **(B)** fungal and Pearson’s correlation analysis of **(C)** bacterial and **(D)** fungal with soil variables. “*,” “**,” and “***” indicate a significant difference at the *p* < 0.05, *p* < 0.01, and *p* < 0.001 level, respectively. Control, no treatment; Alfalfa, alfalfa cultivation; Perilla, perilla cultivation.

The first two axes of the RDA model accounted for 50.92% and 37.59% of the total variance in the bacterial and fungal communities, respectively (Figures 7A,B). The degree of influence of environmental factors on the bacterial community was as follows: EC (R^2^ = 0.8976, *p* = 0.001) > pH (R^2^ = 0.8651, *p* = 0.001) > available phosphorus (R^2^ = 0.5346, *p* = 0.031) > available potassium (R^2^ = 0.5350, *p* = 0.051) > available nitrogen (R^2^ = 0.2823, *p* = 0.223) > soil organic matter (R^2^ = 0.2581, *p* = 0.262; Supplementary Table S1). On the other hand, pH (R^2^ = 0.6504, *p* = 0.002) and EC (R^2^ = 0.4233, *p* = 0.029) were the key chemical factors influencing soil fungal communities (Supplementary Table S2).

The Pearson correlation analysis between bacterial phylum levels and soil environmental factors showed that the relative abundance of the *Actinobacteriota* was significantly positively correlated with EC (*p* < 0.01) and acid phosphatase (*p* < 0.01) and negatively correlated with pH (*p* < 0.01). The relative abundance of *Proteobacteria* was significantly and positively correlated with EC (*p* < 0.01), available potassium (*p* < 0.01), and acid phosphatase (*p* < 0.001). In addition, *Acidobacteriota* was significantly negatively correlated with EC (p < 0.001), available potassium (*p* < 0.05) and acid phosphatase (*p* < 0.001; Figure 7C). In terms of fungi, the relative abundance of *Ascomycota* was significantly positively correlated with urease (*p* < 0.05), which in turn was significantly negatively correlated with sucrase (*p* < 0.05). *Basidiomycota* was significantly negatively correlated with available phosphorus (*p* < 0.05). The relative abundance of *Mortierellomycota* was significantly positively correlated with sucrase (*p* < 0.05), while it was significantly negatively correlated with both AK (*p* < 0.05), LAC (*p* < 0.001) and acid phosphatase (*p* < 0.05; Figure 7D).

### Predictive analysis of microbial community function

**Figure 8:**
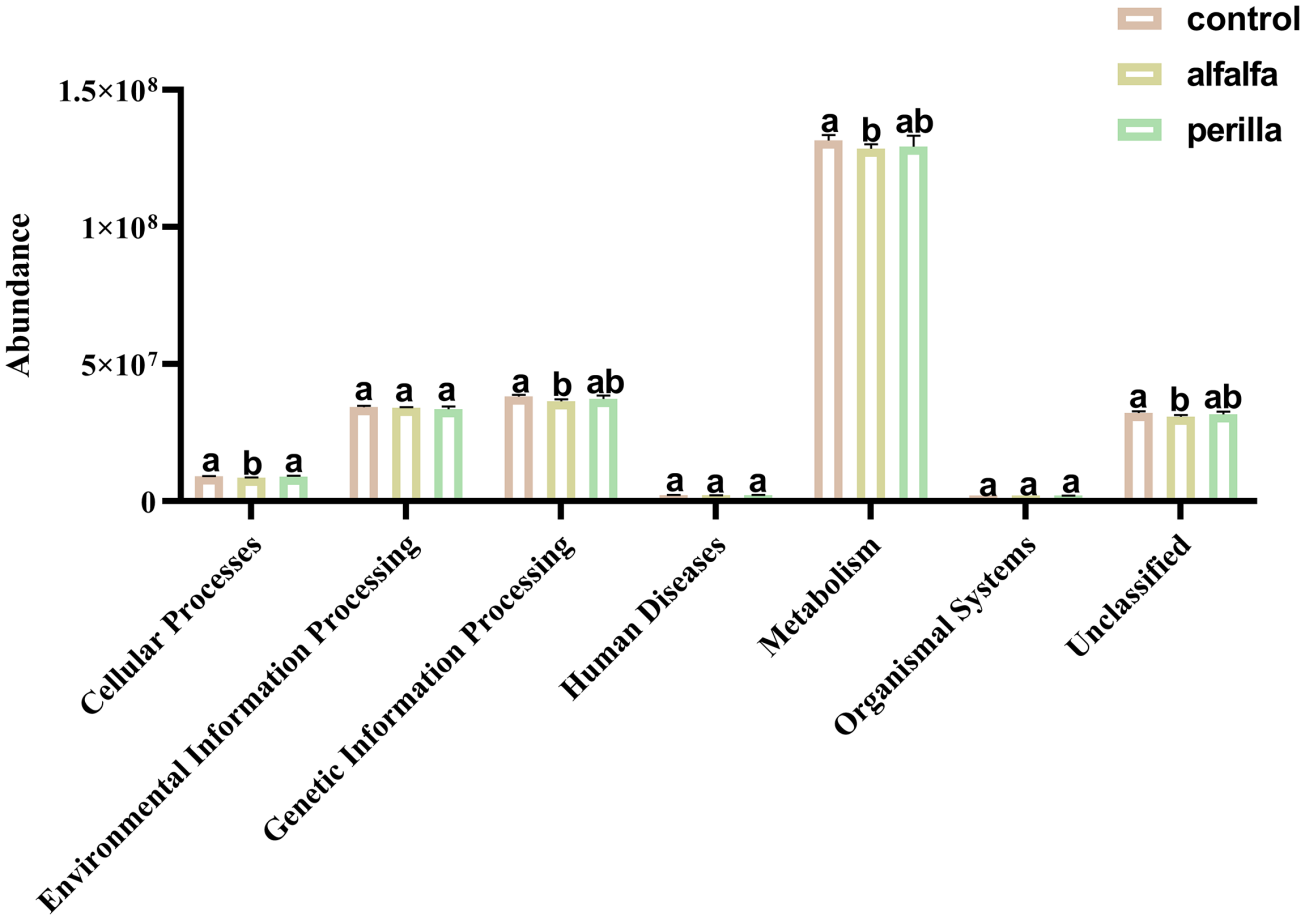
Soil bacterial function prediction in different treatments (Hierarchy level 1). Different letters indicate a significant difference at the *p* < 0.05 level. Control, no treatment; Alfalfa, alfalfa cultivation; Perilla, perilla cultivation.

There are six main types of primary functional layers, including Cellular Processes, Environmental Information Processing, Genetic Information Processing, Human Diseases, Metabolism, and Organismal Systems (Figure 8). Further, 41 secondary functional layers were found, and the top 20 with the highest abundance are shown in Supplementary Figure S6. Among them, the abundance of the alfalfa group in Carbohydrate Metabolism, Replication and Repair, and Energy Metabolism pathways was significantly lower than that of the control group (*p* < 0.05). Functional prediction analysis of each group of fungal communities was carried out by FUNGuild. The top 10 fungal functional groups are shown in Table 1. In this result, Endophyte/Litter Saprotroph/Soil Saprotroph/Undefined Saprotroph was significantly higher in the control group than in the other two groups (*p* < 0.05). On the contrary, Animal Pathogen/Endophyte/Lichen Parasite/Plant Pathogen/Wood Saprotroph in the control group was also significantly lower than that in the other two groups (*p* < 0.05). In addition, Fungal Parasite/Undefined Saprotroph was significantly higher in the alfalfa group than that in the control group (*p* < 0.05).

**Table 1:** Soil fungal function prediction in different treatments.

Name	Control	Alfalfa	Perilla
Undefined saprotroph	27.93 ± 8.616a	23.49 ± 2.082a	24.59 ± 13.84a
Unknown	18.43 ± 2.237a	19.45 ± 2.544a	17.4 ± 2.467a
Endophyte/litter saprotroph/soil saprotroph/undefined saprotroph	18.99 ± 4.221a	9.28 ± 1.8b	7.712 ± 2.656b
Fungal parasite/undefined saprotroph	4.517 ± 0.978b	7.056 ± 2.289a	5.715 ± 0.551ab
Plant pathogen	2.863 ± 0.218a	6.094 ± 0.165a	8.212 ± 7.612a
Animal pathogen/dung saprotroph/endophyte/epiphyte/plant saprotroph/wood saprotroph	5.284 ± 0.900a	2.874 ± 0.503a	3.407 ± 2.004a
Plant pathogen-wood saprotroph	2.665 ± 1.145a	5.627 ± 2.018a	1.6 ± 0.095a
Animal pathogen/endophyte/lichen parasite/plant pathogen/wood saprotroph	0.3119 ± 0.317c	6.611 ± 0.762a	2.136 ± 0.881b
Ectomycorrhizal	0.335 ± 0.213a	0.2268 ± 0.085a	7.087 ± 6.917a
Fungal parasite/plant pathogen/plant saprotroph	0.02696 ± 0.018a	0.1099 ± 0.056a	6.544 ± 12.850a

Control, no treatment; alfalfa, alfalfa cultivation; perilla, perilla cultivation. Different letters indicate a significant difference at the *p* < 0.05 level.

### Ginseng root biomass and root activity

**Figure 9:**
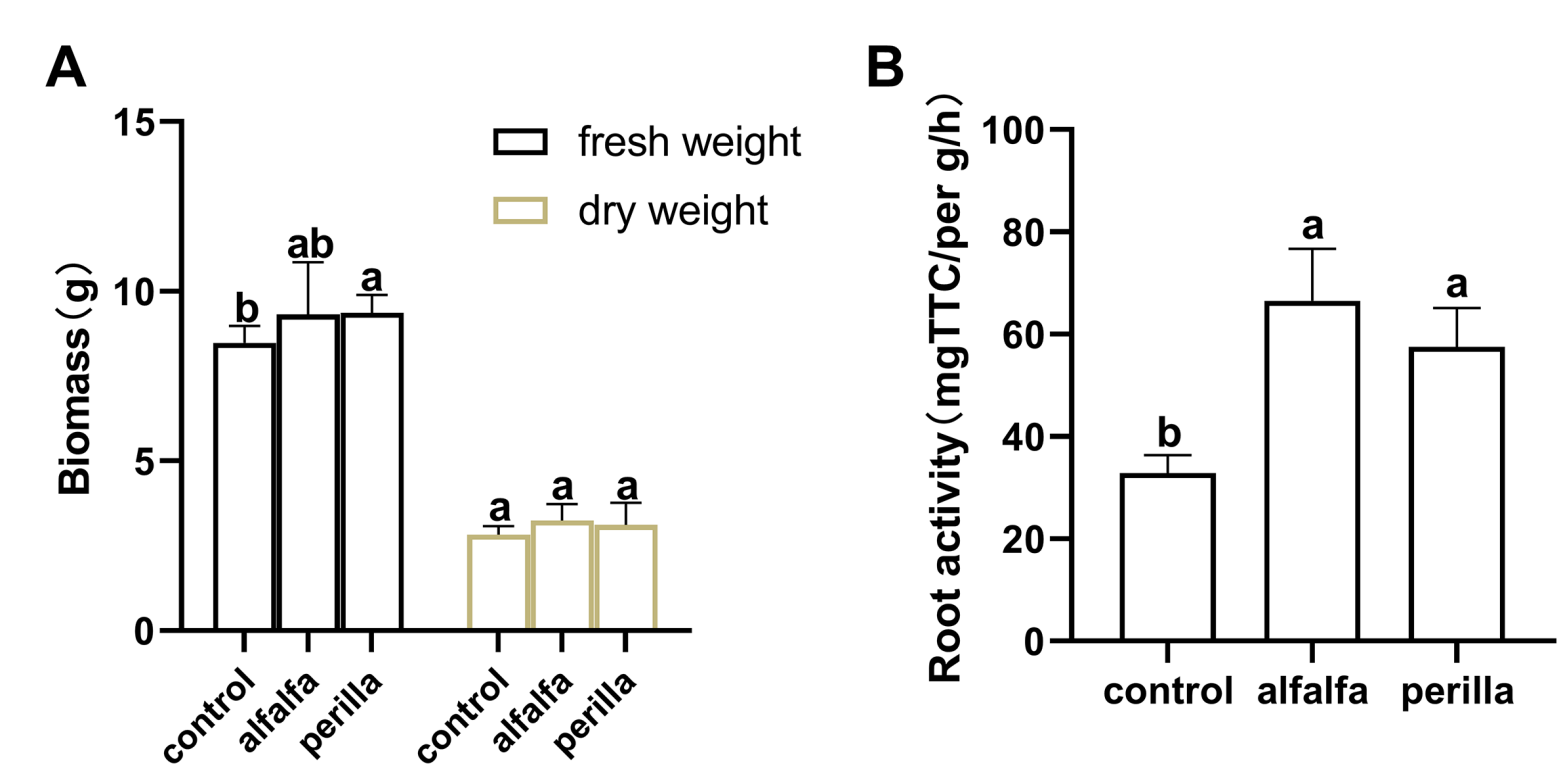
**(A)** Ginseng root biomass and **(B)** root activity in different treatments. Different letters indicate a significant difference at the *p* < 0.05 level. Control, no treatment; Alfalfa, alfalfa cultivation; Perilla, perilla cultivation.

We conducted a pot experiment to detect root biomass and root activity of ginseng cultivated in different treatment groups of soil. After sampling, the biomass of fresh and dry root was determined (Figure 9A). The fresh weight of ginseng root in perilla group was significantly higher than that in control group, but there was no significant difference between the other groups. The root activity of ginseng cultured in soil improved by 1 year-cultivation of alfalfa and perilla was significantly higher than that of untreated soil (control; *p* < 0.05; Figure 9B).

## Discussion

The long term ginseng cultivation can lead to severe soil sickness, and crop rotation is an effective agricultural management to mitigate soil sickness. In the context of the ban on ginseng cultivation in deforestation and the limited land suitable for ginseng cultivation, finding a suitable crop rotation strategy has become an essential task for the sustainable development of the ginseng industry. In this study, we conducted one-year cultivation of alfalfa and perilla on ginseng cultivation soil in northeast China to analyze the effects of both cultivations on ginseng cultivation soil.

### Alfalfa and perilla affected the physicochemical characteristics and enzymatic activities of ginseng cultivation soils

Environmental variables are important indicators for assessing soil quality (Schloter et al., 2003; Chen et al., 2020). Long-term ginseng cultivation can lead to soil acidification, while the heavy use of chemical fertilizers during cultivation can also cause the soil to be susceptible to salinization (Shi et al., 2009). Soil pH has a great influence on the growth and development of ginseng. Previous studies have shown that ginseng is generally suitable for growing in soil with a pH value of 5.0 to 6.5 (Li, 1995). In the soil with strong acidity, ginseng is more susceptible to external stress (You et al., 2015; Wang et al., 2019). In this study, we found that the pH of ginseng cultivation soil increased significantly, while soil salinity (indicated by EC) decreased significantly after 1 year of perilla cultivation. These results indicated that perilla cultivation could effectively improve soil acidification and salinization of ginseng cultivation. The alfalfa cultivation had no significant effect on soil acidity, but it may aggravate soil salinization. The critical difference between these two plants may be related to their differences in root exudates, root associated microbiome and enzyme activities. It is possible that more K ions were solubilized in alfalfa root zone. Although there are few reports on ginseng cultivation and soil salinization, the positive effects of perilla on the pH and EC of ginseng cultivation soils are of interest. Previous studies suggested that high nutrient concentration is the ideal growth condition for ginseng (Sun et al., 2017). This study focused on changes in available nutrients and soil organic matter. Alfalfa and perilla cultivation significantly increased the contents of available nitrogen and available potassium in the soil, and perilla cultivation also considerably increased soil organic matter (Figures 1C,D,F). Previous reports also suggested that alfalfa could improve nitrogen availability in the soil (Bhandari et al., 2020; Samaddar et al., 2021). These results indicated that both plants had positive effects on soil available nutrient pools of ginseng cultivation. It is worth emphasizing that alfalfa and perilla exerted opposite effects on soil EC in this study. Moreover, perilla had a more positive effect on soil EC and organic matter compared to alfalfa. These results will be useful for the future development of fertilization strategies for ginseng rotation systems.

Soil enzymes are derived primarily from soil microorganisms and play an essential role in decomposing organic substrates and biogeochemical cycles (Zhao et al., 2018). Soil urease plays a vital role in the nitrogen cycle by catalyzing the hydrolysis of urea in the soil to produce carbon dioxide and ammonia (Mobley and Hausinger, 1989; Cai et al., 2015). Soil acid phosphatase activity directly affects the hydrolysis process of soil phosphorus compounds. Thus, the increase in soil pH and available nitrogen after perilla cultivation may be related to the increased activity of urease. Both plants are beneficial for phosphorus biotransformation in ginseng cultivation soils. Soil sucrase catalyzes the release of fructose and glucose from sucrose to provide carbon sources for plants and microorganisms (Antonious, 2003). Moreover, the cultivation of alfalfa and perilla significantly increased laccase activity, and laccase has an essential role in forming soil humus and organic matter (Theuerl and Buscot, 2010; Park et al., 2021). Soil catalase can drive the decomposition and transformation of peroxides in soil (Mobley and Hausinger, 1989; Zhu et al., 2021). However, the sucrase activity of the perilla group was lower than that of the untreated ginseng cultivation soil (Figure 2C). In short, alfalfa and perilla cultivation changed the physicochemical properties and enzyme activities of soil under ginseng cultivation to varying degrees, indicating that alfalfa and perilla cultivation may potentially improve soil properties by affecting material circulation and energy metabolism. Moreover, unlike alfalfa, perilla had a more positive effect on soil urease and catalase.

### Alfalfa and perilla affected the microbial communities of ginseng cultivation soils

Plants greatly influence soil microbial communities, and different plant species recruit specific microbial groups (Berendsen et al., 2012). We found that the cultivation of alfalfa and perilla did not significantly change the alpha diversity indexes of soil bacterial communities in ginseng cultivation (Figure 3A). However, for soil fungal communities, perilla cultivation significantly increased the Shannon index, ace index, and chao1 index (Figure 3B). Therefore, these two plants could not affect the diversity and richness of bacterial microbial community in ginseng cultivation soil. Compared with alfalfa, perilla may cause strong fungal microbial selection, increase the diversity and richness of the fungal community in ginseng cultivation soil, and thus improve the ecological environment of ginseng cultivation soil.

The relative abundance of *Actinobacteriota* and *Proteobacteria* increased significantly after 1 year of cultivation of alfalfa, the three most abundant bacterial phyla in the soil, while the relative abundance of *Acidobacteriota* decreased. Interestingly, the cultivation of perilla did not change their abundance (Supplementary Figure S2). *Proteobacteria* was also one of the dominant bacteria in each group of soils. *Proteobacteria* exhibit extreme metabolic diversity, and their members are essential for soil nutrient cycling, mainly in organic phosphate solubilization and soil nitrogen fixation (Long et al., 2018; Ren et al., 2018). *Actinobacteriota* contains a variety of organic matter-degrading bacteria that can maintain high levels of carbon sources in the soil and are critical to the soil carbon cycle (Samaddar et al., 2019). This is why all groups had a higher proportion of *Actinobacteriota* in the soil. It is worth noting that after 1 year of perilla planting, the content of organic matter in soil was significantly higher than that in untreated soil, but the abundance of *Actinobacteriota* in the two groups was not significantly different. This result suggests that there are other potential reasons for the increase in organic matter in Perilla planting soil. In addition, *Acidobacteriota* can also degrade organic matter in the apoplastic matter and maintain an effective nutrient supply to the soil (Ward et al., 2009). Among the three phyla with the highest fungal community abundance, cultivation of alfalfa and perilla only significantly reduced the relative abundance of *Mortierellomycota* (Supplementary Figure S3). In previous studies, *Mortierellomycota*, as saprophytic fungi, played a leading role in the decomposition of soil organic matter (Cong et al., 2020). The decrease in *Mortierellomycota* abundance may be due to the effect of alfalfa and perilla cultivation on soil properties and the special rhizosphere exudates. The accumulation of pathogens in the soil is an essential factor causing soil sickness and continuous cropping disorder. The function of *Ilyonectria* taxa has yet to be fully understood and needs to be further verified. So far, the *Ilyonectria* isolates that have been reported to infect ginseng are divided into four species: *I. robusta*, *I. mors-panacis*, *I. panacis*, and *I. crassa* (Cabral et al., 2012; Farh et al., 2018). *Ilyonectria* may be associated with a variety of soil-borne diseases of ginseng, and its reduced abundance has significant implications for soil health (Liu et al., 2019; Guan et al., 2020; Bischoff Nunes and Goodwin, 2022). It is noteworthy that the cultivation of alfalfa and perilla can effectively reduce the relative abundance of the fungal genus *Ilyonectria* in soil (Supplementary Figure S4; Farh et al., 2018). The possible reason is that the planting of two plants can effectively interrupt the life cycle of this potential pathogen, which has important significance for the sickness of ginseng cultivation soil.

LEfSe tool was used to analyze bacterial and fungal community. The specialized communities that represent microbial communities with statistically significant differences are referred to as indicator microbes (Yan et al., 2021a). LEfSe identified microbial taxa with LDA scores of 3.5 or above. In our results, *Actinobacteriota* and *Chloroflexi* are indicators of control group bacteria. *Proteobacteria*, *Bacteroidota*, and *Acidobacteriota* are the indicator bacteria of the alfalfa group. In the perilla group, class *Vicinamibacteria* and family *Bacillaceae* are indicator microbial taxa (Figure 6A). These results also demonstrated that alfalfa and perilla cultivation changed the bacterial community in ginseng cultivation soil. In Lefse analysis of fungal communities, only *Mortierellomycota* was also screened as an indicator of the control group at the phylum level (Figure 6B). The PCoA and ANOSIM analysis results in this study provide ample evidence that alfalfa and perilla caused profound changes in the composition and structure of microbial communities in ginseng cultivation soil (Figure 5).

To sum up, the microbial communities of ginseng cultivation soil with 1 year of cultivation of alfalfa and perilla were significantly different from those of untreated soil. Cultivation of alfalfa and perilla may modify soil material cycling and energy flow by altering the abundance of some microbial taxa in the soil. In addition, the two plants had different effects on the microbial community, which was related to the different metabolic characteristics of the plant roots (Bi et al., 2021). These changes in microbial communities reflect the potential of both plants, especially perilla, to be applied in alleviating soil sickness in ginseng cultivation.

### Correlations between microbial community structure and soil properties

The close interaction between soil environmental factors and microbial communities has been well-documented by numerous studies (Guo et al., 2019; Neupane et al., 2021). The RDA results showed that soil properties contributed more than 50% and 37% to the alteration of bacterial and fungal microbial community composition, respectively (Figures 7A,B). This result suggests that these soil properties play a dominant role in constructing microbial community structures in ginseng cultivation soils. Pearson correlation analysis showed that soil variables such as pH, available nutrients, and soil enzyme activity had significant positive or negative correlations with the dominant microbial community (Figures 7C,D). Moreover, the ecological functions of these dominant microbial phyla in soils have been well-reported (Yan et al., 2021a).

*Actinobacteriota* was positively correlated with EC and acid phosphatase but negatively correlated with pH. *Proteobacteria* was positively correlated with EC, available potassium, and acid phosphatase. On the contrary, *Acidobacteriota* was negatively correlated with these three environmental factors (Figure 7C). These results indicate some critical environmental factors, especially EC and acid phosphatase, have essential effects on bacteria communities in ginseng cultivation soil. The environmental factors significantly correlated with the dominant phylum of fungi, especially *Mortierellomycota*, were mainly concentrated in soil enzyme activity (Figure 7D). These results indicate that different microorganisms are not equally sensitive to environmental factors. On the other hand, after 1 year of alfalfa and perilla cultivation, dominant bacteria and fungi showed different growth patterns in the soil and played an important role in improvement. The cultivation of two kinds of plants changed the soil environment of ginseng cultivation soil. It provided a different living environment for soil microorganisms, including the difference in pH and available nutrient content, which led to the formation of different microbial networks in the soil. Therefore, alfalfa and perilla may be beneficial for soil health and ecosystem sustainability of ginseng cultivation soils.

Finally, we performed a 6-week pot experiment in which ginseng was grown in different treated soils, and their root activity and biomass were analyzed. The growth and activity of roots directly affect the nutrient level and yield of plants. The root is also an essential medicinal part of ginseng. Compared with untreated ginseng cultivation soil, root activity of 2-year-old ginseng seedlings was significantly improved after 1 year of cultivation with alfalfa and perilla. Meanwhile, the fresh weight of ginseng root in the perilla group was significantly higher than that in untreated soil. There are important interactions among plant roots, soil microorganisms, and soil properties, which are of great significance to nutrient cycling, soil enzymes, and microbial community (Feng et al., 2019). This result further suggests the effect of alfalfa and perilla cultivation on soil properties, enzyme activities, and microorganisms. These factors may favor nutrient transformation and root growth.

## Conclusion

The effects of the two plants on EC were the opposite. In addition, they all increased the available nitrogen, available potassium, acid phosphatase, and laccase of the soil, but only perilla significantly increased the pH, soil organic matter, and urease. Perilla cultivation significantly increased the richness and diversity of the fungal community but had no significant effect on the bacterial community. Cultivation of alfalfa and perilla significantly altered microbial community composition and reduced the abundance of ginseng pathogenic fungus *Ilyonectria* in soil. Generally, perilla has a better improvement effect on soil for ginseng cultivation than alfalfa. Finally, pot experiments showed that the improved soil could significantly improve root activity.

## Data availability statement

The datasets presented in this study can be found in online repositories. The names of the repository/repositories and accession number(s) can be found at: https://www.ncbi.nlm.nih.gov/, PRJNA820351 and https://www.ncbi.nlm.nih.gov/, PRJNA820493.

## Author contributions

XB and XS: conceptualization. YZ, KZ, and XY: data curation. LZ: investigation and supervision. XB and QL: validation, and writing-review and editing. XB and XY: writing - original draft. All authors contributed to the article and approved the submitted version.

## Funding

This research work was supported by the Project of Jilin Provincial Development and Reform Commission (2021C007), the Horizontal Research Project of Jilin Medical University (2021-HX002), the Science and Technology Research Project of Education Department of Jilin Province, China (JJKH20230549KJ), and Jilin Medical University Doctoral Research Startup Fund Project (JYBS2021031LK, JYBS2021026LK, and JYBS2021032LK).

## Conflict of interest

The authors declare that the research was conducted in the absence of any commercial or financial relationships that could be construed as a potential conflict of interest.

## Publisher’s note

All claims expressed in this article are solely those of the authors and do not necessarily represent those of their affiliated organizations, or those of the publisher, the editors and the reviewers. Any product that may be evaluated in this article, or claim that may be made by its manufacturer, is not guaranteed or endorsed by the publisher.

## Footnotes

^1^ http://www.arbsilva.de

^2^ http://unite.ut.ee/index.php
