# Supplementary material for: Potential of Medicago sativa and Perilla frutescens for overcoming the soil sickness caused by ginseng cultivation
Source: Front Microbiol. 2023 Apr 5;14:1134331. doi: 10.3389/fmicb.2023.1134331 (PMC10113677; doi:10.3389/fmicb.2023.1134331)
Supplement: Supplementary file 1 [file Data_Sheet_1.docx]

**Potential of *Medicago sativa* and *Perilla frutescens* for overcoming the soil sickness caused by ginseng cultivation**

Xingbo Bian ^a^, Xiaohang Yang ^a^, Kexin Zhang ^a^, Yiru Zhai ^a^, Qiong Li ^b^, Lianxue Zhang ^c^ and Xin Sun ^a*^

1. Jilin Medical University, Jilin, China
2. Jilin Ginseng Academy, Changchun University of Chinese Medicine, Changchun, China
3. College of Chinese Medicinal Materials, Jilin Agriculture University, Changchun, China


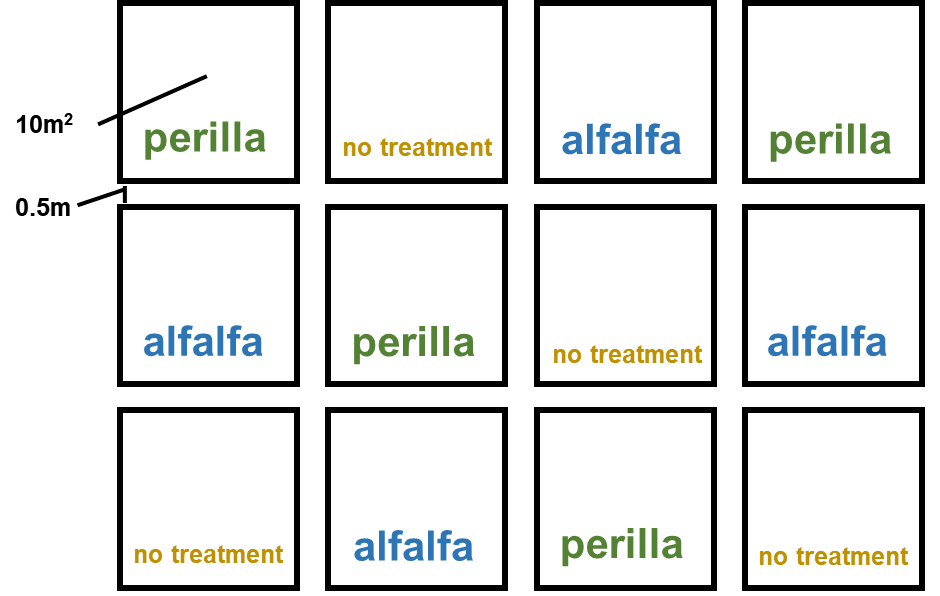


**Fig. S1.** The plots distribution details.

**Fig. S2.** Relative abundances of the dominant bacterial phyla. Different lowercase letters indicate significant differences between the different groups within the same microbial taxa (P＜0.05).

**Fig. S3.** Relative abundances of the dominant fungal phyla. Different lowercase letters indicate significant differences between the different groups within the same microbial taxa (P＜0.05).

**Fig. S4.** Relative abundances of *Ilyonectria*.


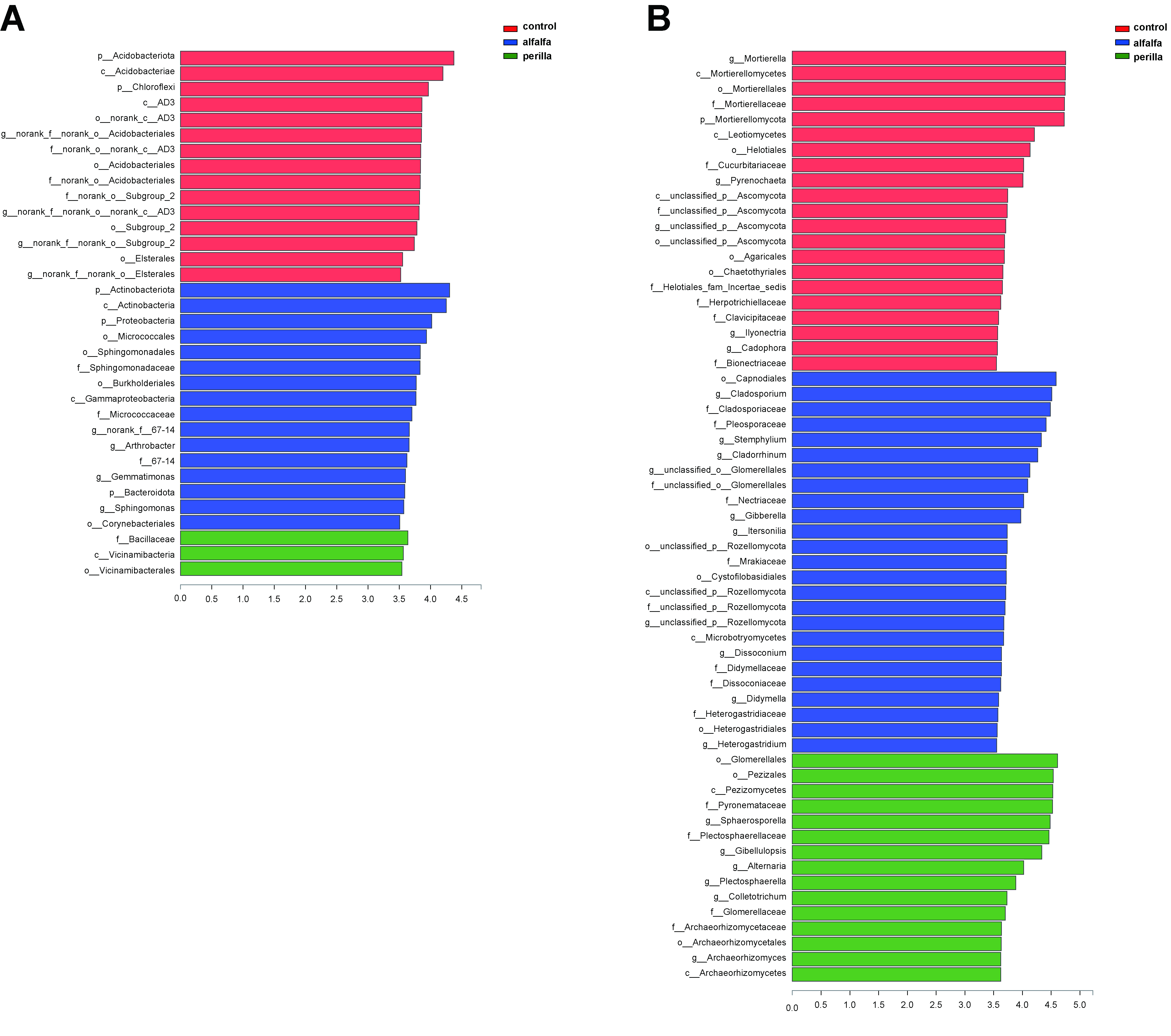


**Fig. S5.** The indicator microorganisms with LDA scores in (A) bacterial and (B) fungi communities associated with each treated soil from the three groups.

**Fig. S6.** Soil bacterial function prediction in different treatments (Hierarchy level 2). Different letters indicate a significant difference at the P < 0.05 level.

|  | **RDA1** | **RDA2** | **R^2^** | **P values** |
| --- | --- | --- | --- | --- |
| **pH** | -0.00258 | -1 | 0.865121658 | 0.001 |
| **EC** | -0.4079 | 0.913025 | 0.897655421 | 0.001 |
| **AN** | -0.99793 | 0.064356 | 0.282305684 | 0.223 |
| **AP** | 0.091744 | -0.99578 | 0.534640505 | 0.031 |
| **AK** | -0.93682 | 0.349814 | 0.534977438 | 0.051 |
| **SOM** | -0.59497 | -0.80375 | 0.258059275 | 0.262 |

**Table S1.** Component extracted matrix of RDA for soil bacteria community.

|  | **RDA1** | **RDA2** | **R^2^** | **P values** |
| --- | --- | --- | --- | --- |
| **pH** | 0.73603 | -0.67695 | 0.650398 | 0.002 |
| **EC** | -0.80243 | 0.596748 | 0.423321 | 0.029 |
| **AN** | 0.995944 | 0.089975 | 0.391917 | 0.072 |
| **AP** | 0.862148 | -0.50666 | 0.189085 | 0.385 |
| **AK** | 0.889455 | 0.457024 | 0.120135 | 0.716 |
| **SOM** | 0.998351 | 0.057401 | 0.282475 | 0.214 |

**Table S2.** Component extracted matrix of RDA for soil fungi community.
